# Wilson Disease and Alpha1-Antitrypsin Deficiency: A Review of Non-Invasive Diagnostic Tests

**DOI:** 10.3390/diagnostics13020256

**Published:** 2023-01-10

**Authors:** Olivier Guillaud, Jérôme Dumortier, Eduardo Couchonnal-Bedoya, Mathias Ruiz

**Affiliations:** 1 Hospices Civils de Lyon, Hôpital Femme-Mère-Enfant, Centre National de Référence pour la Maladie de Wilson, 69500 Bron, France; 2 Ramsay Générale de Santé, Clinique de la Sauvegarde, 69009 Lyon, France; 3 Hospices Civils de Lyon, Hôpital Edouard Herriot, Fédération des Spécialités Digestives, 69003 Lyon, France; 4 Faculté de Médecine Lyon Est, Université Claude Bernard Lyon 1, 69003 Lyon, France; 5 Hospices Civils de Lyon, Hôpital Femme-Mère-Enfant, Service d’Hépatogastroentérologie et Nutrition Pédiatrique, 69500 Bron, France; 6 Hospices Civils de Lyon, Hôpital Femme-Mère-Enfant, Centre National de Référence pour l’Atrésie des Voies Biliaires et les Cholestases Génétiques, 69500 Bron, France

**Keywords:** Wilson disease, alpha1-antitrypsin deficiency, non-invasive tests, diagnosis

## Abstract

Wilson disease and alpha1-antitrypsin deficiency are two rare genetic diseases that may impact predominantly the liver and/or the brain, and the liver and/or the lung, respectively. The early diagnosis of these diseases is important in order to initiate a specific treatment, when available, ideally before irreversible organ damage, but also to initiate family screening. This review focuses on the non-invasive diagnostic tests available for clinicians in both diseases. These tests are crucial at diagnosis to reduce the potential diagnostic delay and assess organ involvement. They also play a pivotal role during follow-up to monitor disease progression and evaluate treatment efficacy of current or emerging therapies.

## 1. Introduction

Wilson disease (WD) and alpha1-antitrypsin deficiency (AATD) are two rare genetic diseases affecting predominantly the liver and/or the brain, and the liver and/or the lung, respectively [1,2]. The early diagnosis of these diseases is important in order to initiate a specific treatment, when available, ideally before irreversible organ damage, but also to initiate family screening. The diagnosis of AATD is usually relatively straightforward, as it relies on the measurement of a serum A1-antitrypsin (AAT) level that is below normal values, whereas the diagnosis of WD can be challenging, as no single test can confirm or exclude the disease.

Liver histology is not routinely required for establishing the diagnosis of AATD or WD but can help by identifying characteristics consistent with either disease, while at the same time excluding other causes, and also by staging/grading the hepatic involvement. In WD, histological findings from liver biopsies are often non-specific [3,4]. Mild steatosis and mild inflammation are often seen at the early stages. Later, an auto-immune hepatitis-like pattern of injury may be present, with parenchymal mononuclear inflammatory infiltrate and interface hepatitis. Portal and periportal fibrosis progresses to portal–portal bridging and finally mixed macro-/micronodular cirrhosis. In cases of acute liver failure (ALF) due to WD, multi-lobular (sub-massive) necrosis may be identified, almost always superimposed upon cirrhosis. Copper is heterogeneously deposited in the liver in WD, varying from lobule to lobule in early disease and from nodule to nodule in cirrhosis, which may lead to negative histochemical staining results for copper deposition, especially in small samples. Quantitative copper analysis of the liver biopsy can help, particularly in difficult cases: high intrahepatic copper (>250 µg/g dry weight) is in favor of the diagnosis of WD in the absence of chronic cholestasis, whereas in untreated patients, normal hepatic copper content (<50 µg/g dry weight) almost always excludes this diagnosis [4]. In patients with severe AATD, while the intrahepatic histological features are variable and mostly non-specific, the most characteristic feature seen is the presence of PAS-positive diastase-resistant inclusions in the cytoplasm of periportal hepatocytes. Other, non-specific, features are the presence of steatosis (sometimes with features of steatohepatitis), lymphocyte-predominant chronic inflammatory cell infiltrate (mostly portal and lobular inflammation, sometimes mild-to-moderate interface hepatitis or centrilobular inflammation), portal fibrosis, bridging fibrosis, and finally cirrhosis [5,6]. Liver biopsy may be helpful for some patients but is associated with morbidity in 0.5% patients (bleeding or pneumothorax) and mortality in 0.02%, and cannot, therefore, be recommended in all patients [7]. In children, it often requires general anesthesia and one overnight stay to observe the absence of bleeding [8].

## 2. Materials and Methods

In this review, we present the non-invasive tests available or in development for clinicians to establish a diagnosis and assess organ involvement in WD and AATD. PubMed/MEDLINE was searched for reports in English language using the following key words: Wilson disease, alpha1-antitrypsin, diagnosis, histology, imaging, genetic. We considered original articles, reviews, meta-analyses, and book chapters published in the last 30 years.

## 3. Results

### 3.1. Wilson Disease

WD is a rare autosomal recessive inherited copper overload disease caused by ATP7B mutations (coding for ATPase 7B) responsible for impaired biliary copper excretion. It results in the accumulation of copper in various tissues and organs, predominantly in the liver, brain and cornea [9]. Making a diagnosis can be challenging given that the disease presentations vary widely and need a combination of diagnostic tests [10]. For these reasons, delayed diagnoses are common. A composite scoring system referred to as the Leipzig score combines the assessment of clinical and mostly non-invasive laboratory or morphological findings (Kayser–Fleischer rings, neurological symptoms or abnormalities on brain MRI, serum ceruloplasmin, Coombs-negative hemolytic anemia, hepatic and urinary copper levels) with genetic data to aid in the diagnosis of WD (Table 1) [11].

#### 3.1.1. Clinical Features at Onset

- WD generally presents in children and young adults between the second and fourth decade of life [12]. However, younger (<5 years) and older ages (>40 years) have been extensively reported at WD onset [13]. Late-onset WD has been reported for both hepatic or neuropsychiatric forms [13,14,15]. Age per se cannot eliminate the diagnosis of WD, even if the index of suspicion should be higher in children and young adults, and a wider screen should be performed at initial presentation in these patients [16]. It also has to be kept in mind that less than 10% of the WD patients were diagnosed after 45 years [17].
- WD patients can present with a wide range of hepatic, neurological, and psychiatric manifestations, combined in various and unpredictable ways [12]. All patterns of liver disease have been described in WD, in both pediatric and adult populations, including asymptomatic abnormalities in liver biochemistry, hepatic steatosis or hepatomegaly on imaging, acute or chronic hepatitis, cirrhosis and acute liver failure [18]. Dysarthria is the most frequent neurological feature reported in large cohorts and is followed by dystonia, tremor, abnormal gait, parkinsonism, and chorea [13]. Dysarthria, dystonia, tremor, and parkinsonism can be the sole initial manifestations of WD. Psychiatric manifestations vary from mood disorders (including depression and bipolar disorders), to personality disorders (including antisocial behavior and sexual disinhibition), and, finally, cognitive impairment [12].
- A Coombs-negative hemolytic anemia and ophthalmological findings such as Kayser–Fleischer ring (KFR) and sunflower cataract are classical non-hepatic/non-neuropsychiatric manifestations seen in WD. Non-immune hemolytic anemia occurs in 4–10% of cases and is highly suggestive of WD when associated with unexplained liver disease or movement disorders [16,19,20] but may also precede other symptoms [21]. The presentation of an unexplained Coombs-negative hemolytic anemia in a child or young adult should always be considered as a case of WD unless proved otherwise. In patients with severe liver failure, marked hemolysis is frequently seen (in around 50–80% of cases [22,23]) and may result from increased non-ceruloplasmin-bound copper released from impaired hepatocytes [20]. KFR consists of a ring-shaped copper deposit in the internal corneal layer of the Descemet’s membrane, seen on slit lamp examination in 90% of neurological presentations, 36–62% of hepatic presentations, and is highly suggestive of WD [24]. KFR can be visible as a yellowish-green or golden-brown discoloration at the periphery of each cornea, distinct from the underlying iris. KFR confirms the presence of excess free copper in the bloodstream but is not pathognomonic for WD, as it may occur in any disorder with impaired biliary copper excretion such as chronic cholestasis. Sunflower cataract is a rare but also characteristic sign, where copper deposits are situated under the lens capsule. Notably, sunflower cataracts do not impact visual acuity and can be reversible after anti-copper treatment [25].
- In non-diagnosed or undertreated women of child-bearing age, infertility or repeated miscarriages are often seen [4,13]. Other rare non-hepatic/non-neuropsychiatric manifestations may be present at the time of diagnosis or develop later, and include renal abnormalities (Fanconi syndrome, nephrolithiasis), cardiac abnormalities (cardiomyopathy, arrythmia), skeletal abnormalities (osteopenia/osteoporosis), pancreatitis, and hypo-parathyroidism.

#### 3.1.2. Laboratory Tests of Copper Metabolism (Table 2)

- The classical triad of “low ceruloplasmin, low serum copper and increased 24-h urinary copper levels” is usually associated with the diagnosis of WD but may be absent in some cases confirmed by genetic testing, and is present in 16% of healthy heterozygous carriers [13]. Some conditions may be responsible for false positive or false negative results and may lead to an incorrect interpretation of copper balance.
- Ceruloplasmin
- A very low ceruloplasmin level (<0.10 g/L; informative cut-off in Leipzig score) is highly suggestive of WD; intermediate concentrations (0.10–0.20 g/L) are less specific, and up to 15% of patients with neurological presentations have normal concentrations (>0.20 g/L) [11,16]. However, a serum ceruloplasmin concentration (measured immunologically) below 0.14 g/L is reported to have a better positive predictive values than a cut-off of 0.20 g/L for the diagnosis of WD [26]. Ceruloplasmin levels have been found to be reduced in other chronic liver diseases, mostly as a consequence of cirrhosis and impaired liver function, but also in cases of malabsorption, malnutrition, marked renal protein loss, acquired copper deficiency, or other rare, inherited metabolic disorders mimicking WD (Menkes disease, aceruloplasminemia, congenital disorders of glycosylation, Nieman Pick C, MEDNIK syndrome) [27]. Moreover, up to 20% of heterozygous ATP7B carriers have a serum caeruloplasmin level of 0.15–0.19 g/L [28]. Conversely, ceruloplasmin is increased by estrogen, pregnancy and contraceptive pills. Being an acute-phase response protein, it also increases during inflammation or infections. Lastly, there are methodological concerns about the widely used immunological assay for ceruloplasmin, which also detects apo-ceruloplasmin and may overestimate results.
- 24 h urinary copper excretion
- The urinary copper concentration is a relatively simple and sensitive test for the diagnosis of WD but also for the monitoring of the effectiveness of WD treatment [4,29,30,31]. In untreated WD patients, the 24 h copper urinary excretion reflects the amount of non-ceruloplasmin-bound copper in the circulation [32]. Diagnostic cut-offs and ranges depend on the laboratory. In a study of 111 healthy adults from the UK, the mean copper output was 0.34 μmol/24 h (21 μg/24 h) [33]. The literature favors cut-offs >1.6 μmol/24 h (>100 μg/24 h) for the diagnosis of WD [15,29,30,34]. However, multiple studies have shown this cut-off to be too high, particularly in children and asymptomatic siblings. With a cutoff >0.64 μmol/24 h (>40 μg/24-h), urinary copper output has 79% sensitivity and 88% specificity for the diagnosis of Wilson disease in children [35]. Moreover, high urinary copper values could be seen in other types of liver disease (e.g., autoimmune hepatitis, NAFLD, cholestasis, and during acute hepatic failure of any origin). Heterozygotes may also have intermediate levels [36].
- The D-penicillamine challenge test (PCT), which consists of measuring the 24 h urinary copper excretion after a dose of D-penicillamine (usually 1 g/day, either once 1 g or twice 500 mg) has been proposed as a tool for WD diagnosis. In a preliminary study, the measurement of 24 h urine copper excretion after PCT was significantly higher in WD patients (three adults and seven children; mean ± standard deviation 37 ± 5.7 µmol/24 h [2330 ± 360 µg/24 h]) in comparison to 10 heterozygote adults (15.9 ± 3.3 µmol/24 h [1000 ± 210 µg/24 h]) and 26 normal adults (10.2 ± 3.7 µmol/24 h [640 ± 230 µg/24 h]) [36]. This was then validated mostly in children: a cut-off >25 µmol/24 h (1600 µg/24 h) had a good sensitivity and positive predictive value for symptomatic patients, but this cut-off was not sufficiently sensitive in asymptomatic patients with WD [37,38,39].
- Serum copper
- Most of the copper measured as serum copper is contained within ceruloplasmin, which explains why in WD patients serum copper is usually low. However, in cases of severe injury related to WD, high serum copper is frequently seen related to a massive release of hepatocellular copper. When considering the diagnosis of WD in the setting of acute liver failure, the interpretation of conventional copper testing is often misleading, as serum copper is high, 24 h urinary copper excretion very high, and ceruloplasmin levels can be either low, normal, or high. These tests are not as reliable as in chronic WD patients and are less sensitive and specific than other available tests such as hemoglobin, serum transaminases, alkaline phosphatase and total bilirubin [40]. In cases of acute liver failure, the presence of a nonimmune acute intravascular hemolysis with severe coagulopathy, relatively modest elevations of serum aminotransferases, normal or subnormal alkaline phosphatase and progression to renal failure is suggestive of the diagnosis of WD [4,41].
- Non-ceruloplasmin-bound copper (NCC)
- The proportion of non-ceruloplasmin-bound (“free”) copper with respect to total copper is increased in patients with WD. NCC can be calculated in µmol/L by subtracting the ceruloplasmin in g/L multiplied by 47 from the serum copper in µmol/L [42]. Variations in the sensitivity and specificity of ceruloplasmin assays between laboratories preclude the definition of an optimal cut-off at diagnosis and during follow-up [43]. Calculated NCC is not useful for WD diagnosis but may help to monitor response with chelation or zinc therapy [42,44].
- An alternative approach consists of measuring the labile fraction of copper bound to albumin and other peptides called exchangeable copper (CuEXC), which represents the majority of NCC. CuEXC is measured by adding EDTA (that binds to the complex ceruloplasmin–copper) to a serum sample and performing ultrafiltration prior to copper quantification [45]. Levels of CuEXC are higher in animal models of WD, in newly diagnosed symptomatic patients, in non-compliant patients, as well as in patients with extrahepatic involvement, and also correlate with neurological severity [46,47,48,49,50]. Expressed as the percentage of total serum copper, relative exchangeable copper (REC) is a valuable diagnostic test with a high sensitivity and specificity. With a cut-off of 18.5%, REC was able to discriminate WD patients from heterozygous ATP7B carriers, healthy controls and patients with other liver diseases [45,47,48,51]. CuEXC has also shown interesting results to monitor treatment response in animal models and WD patients, but an optimal cut-off has yet to be defined [46,48].
- Other techniques directly measuring NCC, such as copper speciation, have been reported recently and are currently under investigation to assess the treatment efficacy in clinical trials [52].

**Table 2:** Classical features of diagnostic tests for WD.

	Normal	Heterozygotes	Pre-Symptomatic WD	Chronic Hepatic WD	ALF-WD	Neurologic WD
Ceruloplasmin (g/L)	normal (≥0.20)	usually normal 0.15–0.19 in 10–15%	≤0.14 but may be normal	≤0.14 but may be normal	usually ≤0.14 often 0.15–0.19 or ≥0.20	≤0.14 but may be normal
Total copper	normal	normal, sometimes decreased	usually decreased	usually decreased	normal or increased	usually decreased
Exchangeable copper	normal	normal	usually normal *	increased but may be normal	Increased *	increased
REC (%)	<18.5	<18.5	>18.5 sometimes 10–18.5% *	>18.5	>18.5 *	>18.5
24 h urine copper (µg/24 h)	<40	usually <40 sometimes 40–100	usually >40	usually >100 sometimes 40–100	>100 (frequently >1000)	>100
Intrahepatic copper (µg/g dry weight)	absent	absent	probably >50 (LB usually not performed)	usually >250 almost always >50	usually >250 almost always >50	usually >250 almost always >50 (LB usually not performed)
Nonimmune hemolytic anemia	absent	absent	absent	sometimes ≤10%	very frequent >60%	sometimes ≤10%
KF ring	absent	absent	<50%	50%	50%	>95%
Brain MRI abnormalities	absent	absent	<50%	50%	50%	>95%
Number of alleles with ATPase 7B disease-causing mutation	0	1	2	2	2	2
Ceruloplasmin (g/L)	normal (≥0.20)	usually normal 0.15–0.19 in 10–15%	≤0.14 but may be normal	≤0.14 but may be normal	usually ≤0.14 often 0.15–0.19 or ≥0.20	≤0.14 but may be normal
Total copper	normal	normal, sometimes decreased	usually decreased	usually decreased	normal or increased	usually decreased
Exchangeable copper	normal	normal	usually normal *	increased but may be normal	increased *	increased
REC (%)	<18.5	<18.5	>18.5 sometimes 10–18.5% *	>18.5	>18.5 *	>18.5
24 h urine copper (µg/24 h)	<40	usually <40 sometimes 40–100	usually >40	usually >100 sometimes 40–100	>100 (frequently >1000)	>100
Intrahepatic copper (µg/g dry weight)	absent	absent	probably >50 (LB usually not performed)	usually >250 almost always >50	usually >250 almost always >50	usually >250 almost always >50 (LB usually not performed)
Nonimmune hemolytic anemia	absent	absent	absent	sometimes ≤10%	very frequent >60%	sometimes ≤10%
KF ring	absent	absent	<50%	50%	50%	>95%
Brain MRI abnormalities	absent	absent	<50%	50%	50%	>95%
Number of alleles with ATPase 7B disease-causing mutation	0	1	2	2	2	2

Abbreviations: WD, Wilson disease; ALF, acute liver failure; LB, liver biopsy; KF, Kayser–Fleischer; MRI, magnetic resonance imaging. * personal data.

#### 3.1.3. Genetic Testing

*ATP7B* sequencing has an important role in confirming the clinical diagnosis in all patients suspected of having WD but should not delay the initiation of treatment. It is also helpful when initial investigations are inconclusive and for family screening. A great number of mutations have been reported that hamper the proper function of ATPase 7B. More than 900 deleterious variants in the *ATP7B* gene have been identified, with single-nucleotide missense and nonsense mutations being the most common, followed by small deletions, splice site mutations and small insertions [53]. In a genetic study of 181 patients from the UK, two mutations were identified in 98% of the participants when using a combination of Sanger sequencing (including coding regions, splice sites, and promoter regions) and multiplex ligation-dependent probe amplification (for the identification of deletions and duplications) [54]. The most common mutation among patients from Northern and Eastern Europe is His1069Gln, but its frequency varies significantly between countries: 86% of patients in Poland, 19% in UK, and 14% in France [54,55,56]. To date, most of the studies have not established a clear phenotype–genotype correlation regarding initial symptomatic manifestations [57]. Studies on modifier genes are ongoing in order to better understand differences in clinical expression despite similar mutations.

#### 3.1.4. Brain Magnetic Resonance Imaging (MRI)

Brain MRI is the most sensitive neuroimaging method for the diagnosis of neurological WD. More than 90% of WD patients with neurological disease and approximately half of the patients with hepatic symptoms have abnormalities on brain MRI. Symmetric hyperintense signal abnormality in the basal ganglia, thalamus, and/or brainstem in T2-weighted or FLAIR images is characteristic of WD [58]. The face of the giant panda sign, where hyperintense signal abnormality surrounds the red nucleus and substantia nigra, is highly specific for WD but only seen in a minority of patients. In addition, signs of diffuse tissue atrophy and hypointensities in T2/T2* and SWI images in the deep gray matter caused by abnormal iron accumulation are frequently present. In order to assess the severity of the radiological findings, Dusek et al. recently proposed a semiquantitative MRI scale that includes an acute toxicity subscore based on the distribution and severity of these hyperintense signal abnormalities and a chronic damage subscore based on brain atrophy and hypointense signal abnormalities on T2/T2*/SWI [59]. In a validation study that included 39 treatment-naïve patients, the acute toxicity subscore improved with treatment (*p* = 0.02) but did not correlate with the Unified Wilson Disease Rating Scale (UWDRS) score at baseline and after 2 years. The chronic damage subscore correlated with UWDRS at baseline (r = 0.59, *p* = 0.005) and 24 months (r = 0.68, *p* < 0.001). However, a recent report on six children with neurological WD has shown that brain MRI had poor prognostic value in distinguishing reversible from irreversible T2-hyperintense lesions after LT [60]. Therefore, brain changes found on MRI should not preclude LT in these patients because they failed to predict clinical outcomes after LT.

#### 3.1.5. Liver Imaging

All patients with suspected WD should have a liver ultrasound (US) evaluation irrespective of their clinical presentation. Patients with exclusive neurological symptoms have a high rate of liver abnormalities on imaging, and morphological signs of liver cirrhosis are frequently found in these patients [61]. Hepatic steatosis is the most common finding, seen in 35–88% of WD patients. Cholelithiasis is not rare in WD and has been reported in 20–25% of patients [62,63]. Irregular liver edge, reversed portal vein flow, splenomegaly or ascites are signs that suggest the presence of cirrhosis. Computed tomography (CT) and/or MRI can also show signs of liver cirrhosis and portal hypertension (PHT). Multiple hyperechoic and hypoechoic nodular lesions with honeycomb pattern, a perihepatic fat layer, and the absence of caudate lobe hypertrophy in a cirrhotic liver are radiological features often seen in WD patients [62,64]. The occurrence of hepatobiliary malignancies (hepatocellular carcinoma and cholangiocarcinoma) during follow-up is a rare complication of WD [17,65]; however, a semestrial screening with abdominal US should be performed in all patients with cirrhosis. Interestingly, in cases of liver tumor, a higher rate of cholangiocarcinoma than expected is observed, which advocates for a frequent histological assessment [65].

#### 3.1.6. Liver Stiffness Measurement (LSM)

Data regarding LSM are relatively scarce in WD, particularly in newly diagnosed patients [66,67,68,69,70]. Paternostro et al. analyzed the results of LSM measured by transient elastography (TE) in 188 WD patients who had concomitant liver biopsy, 44 of whom had recent WD diagnosis before treatment [67]. LSM was higher in cirrhotic compared to non-cirrhotic patients (11.3 vs. 6.1 kPa, *p* < 0.001). This was even more pronounced in recently diagnosed patients (35.2 kPa vs. 6.4 kPa, *p* < 0.001). The accuracy of an LSM cutoff ≥9.9 kPa for the diagnosis of cirrhosis was better in recently diagnosed (predictive positive value (PPV): 74%, negative predictive value (NPV): 100%) vs. previously diagnosed (PPV: 53%, NPV: 82%) patients. In most WD patients, LSM measured by TE remained stable over time (median follow-up of 46 (24–66) months), while a significant proportion (30.8%) of cirrhotic patients showed improvements of LSM under WD therapy. LSM only worsened in 5.6% of recently diagnosed patients and in 8.5% of previously diagnosed patients. In another recent study, a value >10.45 kPa of liver stiffness measured by two-dimensional real-time shear wave elastography (2D-SWE) was associated with an increased risk of hypersplenism [71].

#### 3.1.7. New Non-Invasive Tests

- ATP7B peptides
- Direct quantification of ATP7B peptides on dried blood spots using immunoaffinity-enriched mass spectrometry has recently been proposed as a novel diagnostic test for WD [72]. This technique identified WD patients with a sensitivity of 92% and specificity of 98% in a retrospective cohort of 264 patients and 150 healthy controls. ATP7B peptide concentrations were below diagnostic cut-offs in 101/107 (94%) with Cp < 10 mg/dL, 70/77 (91%) with Cp 10–20 mg/dL, and 14/16 (88%) with normal serum ceruloplasmin (>0.2 g/L). This suggests that a combination of ATP7B peptide quantification and serum ceruloplasmin is likely to improve diagnostic accuracy. Further studies are needed to validate this new technique and confirm these encouraging preliminary findings.
- Anterior segment optical coherence tomography (AS-OCT)
- AS-OCT could be used for the detection of KFR. In a study of 29 patients with WD, 15 had normal slit-lamp evaluation but abnormal AS-OCT (*p* < 0.001), suggesting that AS-OCT is a more accurate diagnostic tool that could detect significantly more cases of KFR as compared to the slit-lamp examination [73]. This technique could allow more easy recognition of KFR by non-expert ophthalmologists, as well as non-ophthalmologists [74,75,76]. It also can be useful to quantify the KF rings that can be followed over time in patients undergoing treatment to assess its effectiveness.
- Brain magnetic resonance spectroscopy (MRS)
- Brain MRS could potentially serve as a method for detecting early neurological involvement, particularly in pediatric patients with WD [77]. MRS demonstrates that N-acetyl-aspartate (NAA) to creatine (Cr) ratios are consistently reduced in the basal ganglia of patients with neurological involvement and that myo-inositol is increased in patients with portosystemic shunting. In a recent case–control prospective study in a pediatric population, the levels of NAA, Cr and choline (Cho), as well as ratios of NAA/Cho, NAA/Cr, and Cho/ Cr in brain tissue as determined by MRS were significantly lower in 26 WD patients compared to 26 healthy individuals [78]. A more severe decrease in NAA, NAA/Cr, and NAA/Cho was observed in WD patients with mixed neurological and hepatic involvement compared with those with liver-only involvement. MRS was more sensitive than conventional MRI in detecting early changes in brains in children with WD before structural changes became visible on MRI.
- Copper absorption test
- Dynamic assessment of copper metabolism and fluxes in the body can be evaluated with radioactive copper tracer, 64Cu. Abnormal copper metabolism with increased hepatic 64Cu retention, reduced fecal excretion of the radiotracer, and altered 64Cu blood kinetics was found in WD patients and in animal models [79,80]. 64Cu incorporation into ceruloplasmin has demonstrated excellent diagnostic accuracy in patients with WD compared to heterozygous control subjects [81]. Interestingly, dynamic positron emission tomography (PET) analysis with 64Cu tracer was used in a recent animal study involving gene therapy [80]. This study found that gene therapy restored physiological copper metabolism in WD mice, confirming the mechanism of action, and demonstrated the translational potential of 64Cu-chloride PET to explore gene therapy pharmacodynamics in a minimally invasive and sensitive manner in WD patients.

### 3.2. A1-Antitrypsin Deficiency

AATD is a rare inherited autosomal recessive disease predisposing to liver damage in both adults and children, and lung involvement in adulthood [82,83]. It results from a misfolding and polymerization of a mutant alpha1-antitrypsin (AAT) protein within the endoplasmic reticulum of hepatocytes. There are more than 500 different alleles described for the protease inhibitor (Pi) allele in the SERPINA1 gene on the long arm of chromosome 14 [84]. The most common pathogenic mutation leading to this misfolded protein is Glu342Lys (Z allele), and a homozygous mutation of the Z allele leads to PiZZ genotype, which predisposes to liver fibrosis and emphysema [85,86]. Prevalence of the Z allele is 1 in 25 persons, and the PiZZ genotype concerns approximately 1 in 2000 persons in European populations [87]. The S allele leads to milder deficiency and less severe phenotypes [86]. Biochemical and genetic tests can confirm this underdiagnosed disease, and different non-invasive tools are available to assess liver and lung involvement.

#### 3.2.1. Circumstances for Diagnosis

- Lung disease results from loss of function due to a low level of serine protease inhibitor capable of inhibiting neutrophil elastase in the lungs, which can lead to the development of early-onset panlobular basal emphysema. AATD-related lung disease only affects adults and should be considered in all cases of chronic obstructive pulmonary disease (COPD), emphysema, bronchiectasis, or asthma with unsuitable response to bronchodilators.
- Liver damage results from the degradation of accumulated AAT polymers in hepatocytes, through autophagy and endoplasmic reticulum pathways, leading to a proinflammatory and fibrogenic response [86,88]. Liver disease may present in infants as neonatal cholestasis with jaundice, pale stools, and elevated serum conjugated bilirubin; this situation requires the early intervention of a pediatric hepatologist [89]. Later during childhood and adulthood, AATD should be considered in patients with chronic elevation of transaminases or, in case of diagnosis of cirrhosis, PHT [90,91,92]. Adults patients with cirrhosis may also present with hepatocellular carcinoma. Insofar as AATD is now a recognized cofactor in chronic liver diseases such as alcoholic and non-alcoholic fatty liver disease, even in heterozygous state, the diagnosis should be sought in all chronic liver disease [93,94,95,96,97].
- Other rare conditions have been reported in ZZ patients, such as neutrophilic panniculitis, ANCA-vasculitis or chronic kidney disease [86,98].
- Since AATD is a genetic disease transmitted in an autosomal recessive mode, family screening can be proposed to first-degree relatives, and be the mode of diagnosis [82].

#### 3.2.2. Diagnostic Tests

**Figure 1:**
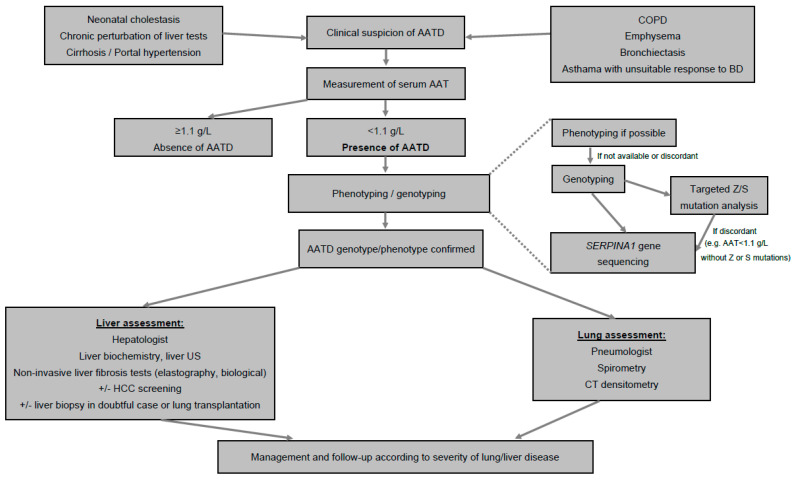
Algorithm for diagnosis of AAT. Abbreviations: AAT, alpha1-antitrypsin; AATD, alpha1-antitrypsin deficiency; COPD, chronic obstructive pulmonary disease; BD, bronchodilators; US, ultrasound; HCC, hepatocellular carcinoma; CT, computed tomography.

- The measurement of serum AAT level is the first test to identify AATD [99]. A concentration < 1.1 g/L is considered low, especially in the absence of inflammatory syndrome, and the most severe phenotypes are usually characterized by AAT concentration < 0.57 g/L. When serum AAT is <1.1 g/L, phenotyping or genotyping tests should be performed in an experienced laboratory (Figure 1) [86,99].
- Phenotyping is performed by iso-electric focusing, which allows one to identify normal (M) and most frequent pathological alleles (Z, S, Mmalton) as well as some rarer ones, according to their electrophoretic mobility, and, therefore, to infer the genotype.
- Sometimes, genotyping of genomic DNA by PCR targeting the most frequent mutant alleles (Z, S) replaces phenotyping, which is controversial because it is a genetic test, and may sometimes wrongly reassure in the absence of Z or S mutation, whereas the AAT level is low due to the presence of other, non-detected (non Z and non S) mutations.
- In doubtful cases, genotyping should be performed by *SERPINA1* gene sequencing in order to confirm the genotype. New approaches are emerging based on blood spot that allow the identification of the Z protein and even a panel of the most frequent phenotypes [100].
- All patients diagnosed with AATD should then be assessed for liver and lung involvement (Figure 1).

#### 3.2.3. Assessment of Liver Disease

- AATD individuals should be referred for clinical evaluation, liver biochemistry, liver ultrasound, and non-invasive fibrosis assessment at the time of diagnosis to detect potential complications and for correct risk stratification [101]. Assessing liver disease severity can be challenging for physicians because fibrosis may develop without severe abnormalities of liver biochemistry, particularly in the case of severe AATD, and because signs of PHT may appear without severe fibrosis [102,103,104,105]. To date, there is no specific treatment available to treat specifically hepatic involvement, and liver transplantation is currently the sole option in cases of decompensated cirrhosis [106,107]. Emerging therapies that may prevent the progression of liver disease are currently under investigation [108,109].
- Clinical evaluation should pay attention to signs of PHT and liver failure.
- Simple blood tests are required, such as for plasma levels of liver enzymes (aspartate amino-transferase (AST), alanine amino-transferase (ALT), gamma-glutamyl-transferase (GGT), alkaline phosphatase (ALP), bilirubin), markers of liver function (prothrombin time (PT) and/or international normalized ratio (INR), albumin, ammoniemia, factor V), and hemogram to identify hypersplenism.
- Abdominal ultrasound (US) should be performed in all patients with AATD to assess liver morphology and search for signs of PHT and focal lesions. In cases with obese patients, CT and/or MRI can be helpful to identify signs of liver cirrhosis and portal hypertension. Screening for hepatocellular carcinoma should be performed every 6 months using US and measurement of serum alpha-fetoprotein in patients with cirrhosis.
- As in all chronic liver diseases, assessment of liver fibrosis severity is a key point in severe AATD. In ZZ patients, classical hepatic risk factors (male sex, age > 50 years, obesity and presence of diabetes) and repeated elevated liver enzymes were associated with significant liver fibrosis [103,110]. Liver biopsy is not necessary for the diagnosis of AATD. It remains the gold standard test for the determination of fibrosis, but is rarely performed in AATD due to its invasive nature, and because non-invasive techniques have been developed over the last years, although there are still limited data available for AATD patients. Liver biopsy remains valuable in discordant situations, when other, concomitant liver disease is associated, and before lung transplantation to assess precisely the liver status [5,102].
- TE is the most widely available technique used routinely in several chronic liver disease to assess liver fibrosis in adults and children by measuring LSM and to estimate steatosis by measuring the controlled attenuation parameter (CAP) [102,111,112,113]. Preliminary studies have found that TE is an easy and repeatable tool to screen for the presence of significant liver fibrosis in AATD adults, and LSM was ≥7.1 kPA (suggesting the presence of significant liver fibrosis) in 18.0–23.6% of adult ZZ patients [94,114]. Clark et al. studied the results of non-invasive tests in comparison to liver histology (METAVIR score) in a population of 94 ZZ adult patients and reported that the best LSM cut-off to identify F ≥ 2 (35.1% patients) and F ≥ 3 (6.4% patients) was 5.45 kPa and 8.45 kPa, respectively [93]. In children, Shneider et al. evaluated TE in a population of cholestatic children including AATD, and showed that this technique was feasible and correlated with liver parameters and PHT. Median LSM was significantly higher in children with PHT (23.8 kPa) compared to patients without PHT (7.8 kPa). In another study, TE had good accuracy in a population of 50 children with AATD, with higher median LSM values in patients with PHT (15.8 kPa) vs. patients without PHT (4.8 kPa), and a threshold of 7.1 kPa (sensitivity 100%, specificity 85%) to detect PHT (personal data). In addition, mean CAP values, an established surrogate of liver steatosis, were found to be higher in Pi*ZZ carriers than in non-carriers, and Pi*ZZ carriers had more severe steatosis than non-carriers, whereas the median body mass index as well as the frequency of obesity and diabetes were similar in both groups [101]. Other techniques, such as acoustic radiation force impulse (ARFI) quantification, two-dimensional shear wave elastography (2D-SWE), and magnetic resonance elastography (MRE), have been assessed in small cohorts of Pi*ZZ individuals and remain to be comprehensively validated [115,116]. MRE, which is a less routinely available technique, seems to be particularly useful in difficult cases such as those involving obesity and allows steatosis quantification.
- In addition to elastography methods, or when these are not available, simple liver biology tests can be used to calculate indirect non-invasive biological fibrosis scores, such as the AST to platelet ratio index (APRI) and the Fibrosis-4 test (FIB-4). Although data are scarce, these non-invasive markers of fibrosis seem to have good accuracy to detect fibrosis in adults and perform as well as transient elastography, whereas they are not as accurate to determine F ≥ 3 [93]. When compared with liver histology, APRI > 0.43 and 0.63 were associated with fibrosis ≥ F2 and ≥F3, respectively, with high specificity (87 and 94%) but low sensitivity (59 and 57%) in adults. In children, recent data suggest that a value > 0.67 (sensitivity 94% and specificity 95%) was associated with significant PHT (personal data). The accuracy of FIB-4 seems to be poorer in adults when compared with liver histology with lower sensitivity (74 and 71%) and specificity (64 and 78%) for cut-offs >1.43 and 1.90, respectively for F ≥ 2 and F ≥ 3 in adults [93]. Other non-invasive laboratory tests (Fibrotest^®^, Fibrometer^®^, ELF^®^) have not been studied in AATD. Similarly to the management of other liver diseases, the use of a combination of a laboratory test and an elastography method to identify and monitor liver fibrosis could be proposed, and in the event of discordant results, a biopsy might be considered [102]. However, such a strategy remains to be specifically validated in AATD patients.

#### 3.2.4. Assessment of Lung Disease

- The severity of lung disease is variable, from asymptomatic to severe, and depends on genotypes and behavior such as smoking. Lung involvement could be treated with AAT augmentation therapy and may require lung transplantation in the most severe patients [86]. Therefore, it is crucial to assess severity at diagnosis and to monitor it [82]. The clinical manifestations of AATD-related lung disease are similar to those of other causes of emphysema, which contributes to underdiagnosis and late diagnosis of the disease. It is characterized by basal panlobular emphysema at an early age, with generally reversible airflow obstruction, usually chronic bronchitis, and bronchiectasis.
- Simple clinical evaluations using the 6 min walk test and health-related quality of life questionnaires should be regularly performed [82]. Pulmonary function tests including spirometry with the forced expiratory volume in the first second (FEV1) and the diffusing capacity of the lungs for carbon monoxide (DLCO) are performed to assess severity during follow-up [82].
- CT densitometry is an imaging method used to assess lung volume and emphysema, and in AATD this provides essential information regarding the presence, distribution and morphology of emphysema but also offers the option to quantify the extent of emphysema. These data, combined with the results of pulmonary function tests, have implications for treatment decisions such as initiation of AAT therapy, or suitability for surgical or endoscopic interventions for reducing lung volume [117]. In addition, the course of the lung density seems to be one of the most specific and sensitive surrogate end-points for evaluation of the therapeutic benefit of augmentation therapy and represents a paradigm imaging biomarker [82,118].

## 4. Conclusions

Non-invasive diagnostic tests are crucial for the diagnosis of rare genetic diseases such as WD and AATD to reduce the diagnostic delay and assess organ involvement. These tests also play a pivotal role during the follow-up of patients by monitoring the effectiveness and tolerability of current treatments and new emerging therapies, such as gene therapy and new molecules, which should be available soon.

## Figures and Tables

**Table 1:** Leipzig score (a scoring system for the diagnosis of Wilson disease).

Parameter		Points
**Specific clinical features**		
Kayser–Fleischer rings (by slit-lamp examination)		
	Present	2
	Absent	0
Neuropsychiatric symptoms suggestive of WD (or typical features on brain MRI *)		
	Present	2
	Absent	0
Coombs-negative hemolytic anemia (+high serum copper)		
	Present	1
	Absent	0
**Laboratory tests**		
24 h urinary copper excretion (in the absence of acute hepatitis)	Normal (<100 µg/d in adults and <40 µg/d in children)	0
	1–2 × ULN	1
	>2 × ULN	2
	Normal but >500 µg/d one day after challenge with 2 × 500 mg D-penicillamine	2
Liver copper quantitative **		
	Normal (<50 µg/d dry weight)	−1
	Up to 5 × ULN	1
	>5 × ULN	2
Rhodanine-positive hepatocytes (only if quantitative copper measurement not available)		
	Absent	0
	Present	1
Serum ceruloplasmin (nephelometric assay, normal: >20 mg/dL ***)		
	Normal	0
	10–20	1
	<10	2
**Mutation analysis (ATP7B)**		
	Disease-causing mutations on both chromosomes	4
	Disease-causing mutations on one chromosome	1
	No disease-causing mutation detected	0
Total score (not available: scores 0)		
**Assessment of the WD diagnosis score**		
4 or more: Diagnosis of Wilson disease highly likely		
2–3: Diagnosis of Wilson disease probable, conduct more investigations		
0–1: Diagnosis of Wilson disease unlikely		

Abbreviations MRI, magnetic resonance imaging; EEG, electroencephalogram; ULN, upper limit of normal; WD, Wilson disease. * Detailed MRI or EEG studies are only needed if neurologic symptoms cannot be excluded with certainty by clinical-neurological examination. ** Liver biopsy is not mandatory for diagnosis and evaluation of neurologic symptoms. Histopathologic assessment of liver was considered to be important for clinical research protocols. *** Other values may apply when ceruloplasmin is measured by the oxidase assay.

## Data Availability

Not applicable.
